# Comparative Genomic Analysis and Data About the Metabolism of the Genus *Sphaerotilus* Provide the First Evidence of Methylotrophic Growth and Reveal Two Strategies of Methanol Oxidation and C1 Compound Assimilation

**DOI:** 10.3390/ijms27125498

**Published:** 2026-06-18

**Authors:** Dmitry D. Smolyakov, Tatyana S. Rudenko, Margarita Y. Grabovich

**Affiliations:** Department of Biochemistry and Cell Physiology, Voronezh State University, 394018 Voronezh, Russia; songolifreya@mail.ru (D.D.S.); ipigun6292@gmail.com (T.S.R.)

**Keywords:** filamentous colorless bacteria, *Sphaerotilus*, methylotrophy, C1 compound assimilation, Calvin–Benson–Bassham cycle, serine cycle

## Abstract

For the first time in this study, the ability for methylotrophic growth on methanol was demonstrated in representatives of the genus *Sphaerotilus*. The analysis of 20 genomes and the physiological verification of genomic predictions regarding C1 compound metabolism were carried out using *Sphaerotilus montanus* HS^T^, *Sphaerotilus hippei* DSM 566^T^, and *Sphaerotilus sulfidivorans* D-501^T^ as model strains. Genes involved in the direct oxidation of methanol to carbon dioxide were identified, including the lanthanide-dependent methanol dehydrogenase XoxF, the NAD-dependent methanol dehydrogenase Mdh2, genes of the tetrahydromethanopterin (H_4_MPT) and tetrahydrofolate (H_4_F) pathways, and the NAD-dependent formate dehydrogenase. In addition, a number of genes associated with C1 assimilation were identified, including genes of the Calvin–Benson–Bassham cycle and the incomplete serine cycle. Experimental data suggest that the bacteria are capable of using two strategies of methylotrophic growth: methanol oxidation via the lanthanide-dependent methanol dehydrogenase XoxF and the H_4_MPT pathway, as well as oxidation via the NAD-dependent methanol dehydrogenase Mdh2 and the H_4_F pathway. Both strategies provide CO_2_ assimilation via the Calvin–Benson–Bassham, but additionally the second strategy demonstrates additional involvement of the incomplete serine cycle in the process of the C1 compounds. A hypothetical model of C1 compound assimilation in representatives of the genus *Sphaerotilus* was constructed.

## 1. Introduction

The group of bacteria *Sphaerotilus–Leptothrix*, which was recently reclassified into a single genus *Sphaerotilus* [1], is represented by filamentous colorless bacteria that are widely known for their ability to oxidize manganese and iron [2,3,4,5,6], forming dense biofilms in natural and anthropogenic aquatic ecosystems [7,8,9,10,11].

Representatives of the genus *Sphaerotilus* are cosmopolitan. This status is due to their high ability to colonize various ecological niches and form dense biofilms, thereby allowing them to become a structural component of microbial communities [9,12,13,14].

With the increasing number of available genome assemblies and new data on genome annotation for representatives of the recently revised genus *Sphaerotilus*, it is evident that these bacteria are characterized by a flexible type of metabolism [15,16]. Genomic analysis demonstrated that, in addition to the ability for chemoorganoheterotrophic growth, this group of bacteria has the potential for autotrophy, molecular nitrogen fixation, dissimilatory nitrate reduction, as well as for lithotrophy using reduced compounds of sulfur, iron, and manganese, thus significantly expanding our understanding of their ecological role [1,6,17].

However, despite the identified metabolic flexibility, data on the ability of representatives of the genus *Sphaerotilus* to grow methylotrophically are currently lacking, therefore making this aspect of metabolism a promising direction for further research.

Methanotrophy and methylotrophy are specific types of metabolism in which C1 compounds (methane, methanol, and other C1 compounds) are used as the primary source of carbon and energy [18,19]. Methylotrophy has remained a key topic in microbiological research since the first methylotrophic microorganisms were described [20]. The metabolism of C1 compounds is characterized by high energy efficiency and provides the cell with both ATP and reducing equivalents (NADH), as well as electrons for the respiratory chain and carbon for the biosynthesis of cellular components [21]. Methylotrophic and methanotrophic bacteria play an important role in the global carbon cycle (greenhouse effect) and the detoxification of natural ecosystems, which determines their ecological and practical significance [22,23,24]. They are widely used for the production of feed protein in agriculture due to the low cost of the resulting products [25].

The process of direct methanol oxidation includes several sequential reactions. At the first stage, methanol is oxidized to formaldehyde by methanol dehydrogenases [20,26]. For prokaryotes, three types of methanol dehydrogenases are known: the pyrroloquinoline quinone (PQQ)-dependent (the cofactor is Ca^2+^) MxaFI, which is a heterotetramer consisting of the MxaF and MxaI subunits and requiring the products of the *mxaACKLGRSBJD* gene cluster [27,28,29,30]; the PQQ-dependent XoxF (the cofactor is La^3+^) [31,32,33]; and the NAD-dependent methanol dehydrogenase Mdh2, which has a low affinity for methanol compared to MxaF/XoxF methanol dehydrogenases [34,35]. In MxaFI/XoxF methanol dehydrogenases, the electrons released during substrate oxidation are transferred from the PQQ cofactor through specific cytochrome carriers to the electron transport chain, whereas the Mdh2 type directly reduces NAD^+^ to NADH [30].

Further oxidation of formaldehyde can proceed via two alternative pathways, the tetrahydromethanopterin (H_4_MPT) and tetrahydrofolate (H_4_F) pathways, with the formation of formate, which is then oxidized to carbon dioxide with the participation of formate dehydrogenase [36,37].

Assimilation of C1 compounds during methylotrophic growth is possible via methylene-H_4_F in the serine cycle, formaldehyde in the ribulose monophosphate (RuMP) cycle, and carbon dioxide in the Calvin–Benson–Bassham cycle [21,38,39,40].

Although studies on the ability of representatives of the genus *Sphaerotilus* to utilize C1 compounds are limited and mainly focus on the analysis of genes associated with carbon dioxide fixation [1,15,41], the range of potentially available C1 substrates includes a wide variety of compounds. Considering that bacteria of the genus *Sphaerotilus* are widely distributed in anthropogenic ecosystems, the study of their potential to use C1 compounds may be important for understanding the mechanisms of competitive interactions and the stability of microbial communities in anthropogenic and natural ecosystems [42,43].

In this study, based on biochemical data and gene expression, a verification of genomic data for a number of pure cultures was carried out, indicating the potential for methylotrophy in representatives of the genus *Sphaerotilus*. The obtained results made it possible to propose a hypothetical model of methanol metabolism and assimilation of C1 compounds in bacteria of the genus *Sphaerotilus*, expanding the understanding of their ecological strategies and their role in anthropogenic and natural ecosystems. The study of new groups of microorganisms capable of methylotrophy will help expand the understanding of consortia that play a key role in the migration and transformation of C1 compounds.

## 2. Results

### 2.1. Analysis of Genes Associated with Methylotrophic Growth

#### 2.1.1. Genes Involved in Methanol Oxidation to Carbon Dioxide

A comprehensive analysis of 20 genomes of representatives of the genus *Sphaerotilus* revealed the absence of genes encoding methane monooxygenase (MMO), which catalyzes the oxidation of methane to methanol.

At the same time, genes encoding methanol dehydrogenases were identified, which indicates the potential ability of these bacteria for methylotrophic growth, where methanol and its derivatives act as sources of carbon and energy.

**Figure 1:**
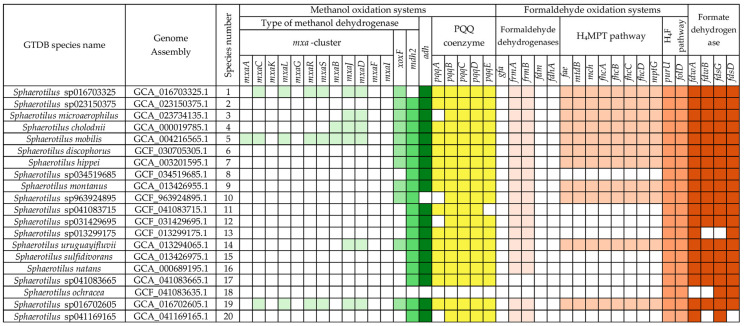
The map of the composition of genes involved in the direct oxidation of methanol to carbon dioxide. Each system is assigned a distinct color indicating the presence of the respective genes. Gene absence is denoted by the lack of coloration. Gene composition: *mxaA*—MxaA protein; *mxaC*—MxaC protein; *mxaK*—MxaK protein; *mxaL*—MxaL protein; *mxaG*—cytochrome c-L; *mxaR*—MxaR protein; *mxaS*—MxaS protein; *mxaB*—MxaB protein; *mxaJ*—MxaJ protein; *mxaD*—MxaD protein; *mxaF*—methanol dehydrogenase (cytochrome c) subunit 1 [EC 1.1.2.7]; *mxaI*—methanol dehydrogenase (cytochrome c) subunit 2 [EC 1.1.2.7]; *xoxF*—lanthanide-dependent methanol dehydrogenase [EC 1.1.2.10]; *mdh2*—methanol dehydrogenase [EC 1.1.1.244]; *adh*—alcohol dehydrogenase [EC 1.1.1.1]; *pqqA*—pyrroloquinoline quinone biosynthesis protein A; *pqqB*—pyrroloquinoline quinone biosynthesis protein B; *pqqC*—pyrroloquinoline quinone synthase [EC 1.3.3.11]; *pqqD*—pyrroloquinoline quinone biosynthesis protein D; *pqqE*—pyrroloquinoline quinone biosynthesis protein E; *gfa*—S-(hydroxymethyl)glutathione synthase [EC 4.4.1.22]; *frmA*—S-(hydroxymethyl)glutathione dehydrogenase [EC 1.1.1.284]; *frmB*—S-formylglutathione hydrolase [EC 3.1.2.12]; *mdo*—formaldehyde dismutase [EC 1.2.98.1]; *fdhA*—glutathione-independent formaldehyde dehydrogenase [EC 1.2.1.46]; *fae*—formaldehyde-activating enzyme [EC 4.2.1.147]; *mtdB*—methylene-tetrahydromethanopterin dehydrogenase [EC 1.5.1.-]; *mch*—methenyltetrahydromethanopterin cyclohydrolase [EC 3.5.4.27]; *fhcA*—formylmethanofuran dehydrogenase subunit A [EC 1.2.7.12]; *fhcB*—formylmethanofuran dehydrogenase subunit B [EC 1.2.7.12]; *fhcC*—formylmethanofuran dehydrogenase subunit C [EC 1.2.7.12]; *fhcD*—formylmethanofuran-tetrahydromethanopterin N-formyltransferase [EC 2.3.1.101]; *mptG*—beta-ribofuranosylaminobenzene 5′-phosphate synthase [EC 2.4.2.54]; *purU*—formyltetrahydrofolate deformylase [EC 3.5.1.10]; *folD*—methylenetetrahydrofolate dehydrogenase (NADP^+^)/methenyltetrahydrofolate cyclohydrolase [EC 1.5.1.5; 3.5.4.9]; *fdwA*—formate dehydrogenase major subunit [EC 1.17.1.9]; *fdwB*—formate dehydrogenase beta subunit [EC 1.17.1.9]; *fdsG*—formate dehydrogenase subunit gamma; *fdsD*—formate dehydrogenase subunit delta [EC 1.17.1.9]. The complete annotation of enzyme genes involved in the pathways is provided in Table S1 (Supplementary Materials).

**Figure 2:**
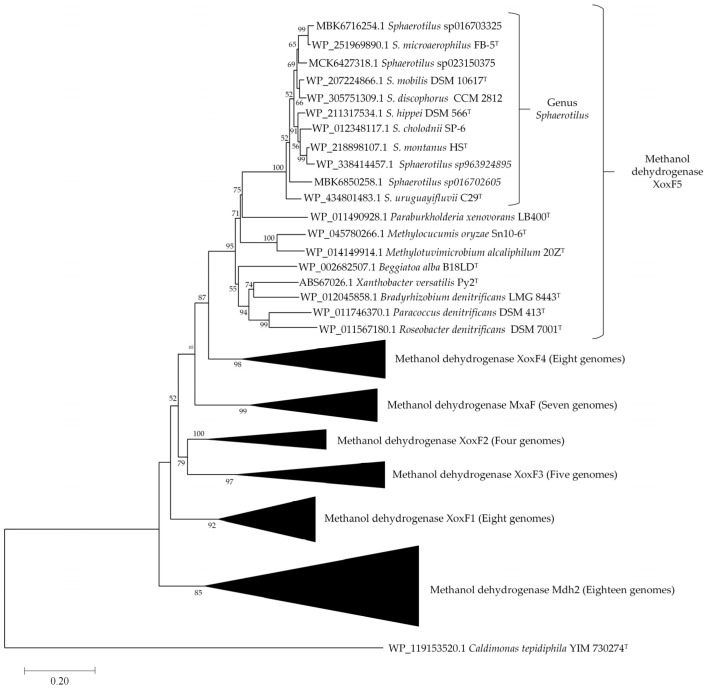
Phylogenetic tree of *Sphaerotilus* methanol dehydrogenases based on MxaF, XoxF1–5, and Mdh2 protein sequences constructed using the minimum evolution method. Mdh2 proteins detected in *Sphaerotilus* were included for completeness and formed a distinct clade separate from XoxF proteins. Numbers at the branch nodes indicate bootstrap values expressed as percentages of 1000 replicates. The phylogenetic analysis included reference sequences with previously established phylogenetic positions and functional characteristics [44,45]. The tree was rooted using the alcohol dehydrogenase of *Calidimonas tepidiphila* YIM 73027^T^ (WP_119153520.1) as an outgroup. The scale bar represents 0.2 substitutions per site. A complete list of reference proteins from other bacterial species used for tree construction is provided in Table S2 (Supplementary Materials).

In 55% of the analyzed genomes of the genus *Sphaerotilus*, genes encoding the PQQ-dependent methanol dehydrogenases XoxF or MxaF were identified, whereas genes encoding the NAD-dependent methanol dehydrogenase Mdh2 were present in 95% of the genomes (Figure 1). Considering the high level of homology between XoxF and MxaF proteins, their differentiation is difficult; therefore, a phylogenetic analysis based on proteins with known functions was performed to distinguish between these types (Figure 2).

Phylogenetic analysis showed that all methanol dehydrogenase (PQQ-associated types) proteins identified in representatives of the genus *Sphaerotilus* cluster exclusively with the XoxF type, while the MxaF clade forms a separate branch and does not include any of the studied proteins from *Sphaerotilus*. Currently, five phylogenetic variants of XoxF are known [21,46]. According to the phylogenetic analysis, *Sphaerotilus* proteins belong to the lanthanide-dependent methanol dehydrogenase group XoxF5 (Figure 2). To further assess the clustering pattern of methanol dehydrogenase proteins obtained using the minimum evolution method, an additional phylogenetic tree was reconstructed using the maximum likelihood approach. The maximum likelihood tree showed a topology consistent with that obtained using the minimum evolution method (Figure S1, Supplementary Materials).

In addition, in several genomes, in the absence of genes encoding the catalytic subunits of MxaFI, individual genes of the *mxa*-cluster (*mxaACKLGRSBJD*), encoding regulatory and accessory proteins associated with MxaFI, were identified. In particular, in genomes 1, 3, 4, 5, 14, and 19, only fragments of this cluster were detected (1—*mxaCLRSJD*; 3—*mxaJD*; 4—*mxaBJD*; 5—*mxaACLRSBJD*; 14—*mxaJD*; 19—*mxaCLRSJD*) (Figure 2).

Thus, phylogenetic analysis and the gene composition of the accessory *mxaACKLGRSBJD* cluster support the conclusion that the PQQ-dependent methanol dehydrogenases identified in *Sphaerotilus* belong to the XoxF5 type.

The synthesis of the universal cofactor of methanol dehydrogenases (XoxF/MxaFI), pyrroloquinoline quinone, is encoded by the *pqqABCDE* gene cluster. A complete set of *pqqABCDE* genes was identified in 70% of the analyzed genomes, while 25% were missing individual genes (*pqqA* or *pqqE*); another 5% of genomes lack genes from this cluster (Figure 1).

Further oxidation of toxic formaldehyde may occur via several enzymatic systems: with the participation of formaldehyde dehydrogenases, formaldehyde dismutase (*mdo*), glutathione-dependent formaldehyde dehydrogenase (*fdhA*), the glutathione-dependent pathway involving S-(hydroxymethyl)glutathione synthase (*gfa*), S-(hydroxymethyl)glutathione dehydrogenase (*frmA*), and S-formylglutathione hydrolase (*frmB*), as well as the H_4_MPT and H_4_F pathways [37,47].

To expand the understanding of the ability of representatives of the genus *Sphaerotilus* to detoxify formaldehyde and to assess the potential role of formaldehyde dehydrogenases in methylotrophic growth, the corresponding genes were analyzed. Genes encoding formaldehyde dismutase (*mdo*) and glutathione-dependent formaldehyde dehydrogenase (*fdhA*), which catalyze oxidation of formaldehyde to formate, were absent in the genomes of *Sphaerotilus*. The process of stepwise oxidation of formaldehyde to formate via hydroxymethylglutathione and formylglutathione cannot be realized in representatives of *Sphaerotilus*, since the *gfa* gene encoding S-(hydroxymethyl)glutathione synthase, which catalyzes the first step of this pathway, is absent in all analyzed genomes (Figure 1). However, the *frmA* and *frmB* genes, which encode S-(hydroxymethyl)glutathione dehydrogenase and S-formylglutathione hydrolase, respectively, are present in many strains.

Genes of the H_4_MPT pathway are marker genes for methylotrophs [18]. Genes of the H_4_MPT pathway (*fae*, *mtdB*, *mch*, *fhcABCD*, *mptG*) were found in 55% of the analyzed genomes. Notably, in representatives of the genus *Sphaerotilus*, a correlation is observed between the presence of the XoxF methanol dehydrogenase gene and genes of the H_4_MPT pathway (Figure 1).

In the absence of the H_4_MPT pathway, the function of formaldehyde detoxification and oxidation is likely carried out by the H_4_F pathway, the genes of which are encoded in all analyzed genomes. In representatives of the genus *Sphaerotilus*, the H_4_F pathway is realized with the participation of the bifunctional enzyme methylenetetrahydrofolate dehydrogenase (NADP^+^)/methenyltetrahydrofolate cyclohydrolase (*folD*), which catalyzes the sequential conversion of methylene-H_4_F to methenyl-H_4_F and then to formyl-H_4_F. Then, formyl-H_4_F is transformed into formate by formyltetrahydrofolate deformylase (*purU*) with the release of H_4_F (Figure 1).

Formate oxidation in all species of the genus *Sphaerotilus* is carried out by the NAD-dependent aerobic formate dehydrogenase (*fdwAB, fdsGD*); however, in some genomes, partial loss of one or two genes is observed (15% of genomes) (Figure 1).

It is noted that the activity of enzymes of the H_4_MPT pathway is several times higher than that of enzymes of the H_4_F pathway, and the activity of the XoxF is higher than that of the Mdh2 [35,48]. The XoxF and the H_4_MPT pathway suggest more efficient oxidation of methanol via formaldehyde to formate (55% of genomes) than the combination of the Mdh2 and the H_4_F pathway (95% of genomes).

The only representative of the genus *Sphaerotilus* lacking a potential for methylotrophic growth is genome 18, due to the absence of genes encoding methanol dehydrogenases and the *pqqABCDE* cluster.

#### 2.1.2. Analysis of Genes for C1 Compound Assimilation During Methylotrophic Growth

Assimilation of C1 compounds during methylotrophic growth can occur via three pathways: the RuMP cycle, the serine cycle, and the Calvin–Benson–Bassham cycle [40].

**Figure 3:**
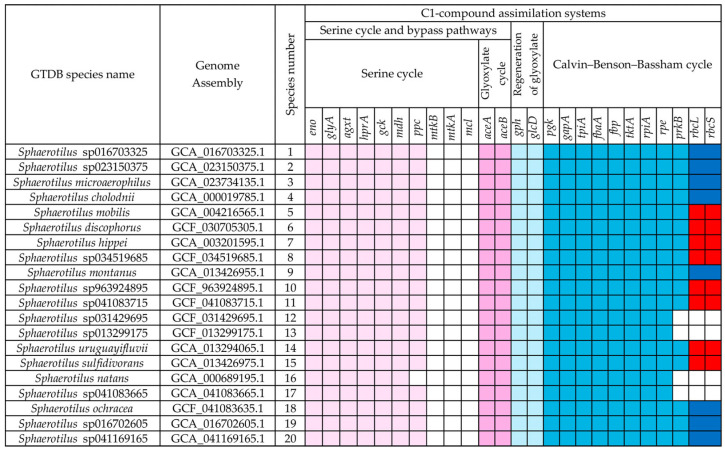
The map of the composition of genes involved in the assimilation of C1 compounds. Each system is assigned a distinct color indicating the presence of the respective genes. Gene absence is denoted by the lack of coloration. For *rbcLS* genes, red and blue indicate RuBisCO forms IC and II, respectively. The cells were merged to indicate RuBisCO form II, which contains only the large subunit [1]. Gene composition: *eno*—enolase [EC 4.2.1.11]; *glyA*—glycine hydroxymethyltransferase; *agxt*—alanine-glyoxylate transaminase/serine-glyoxylate transaminase/serine-pyruvate transaminase [EC 2.6.1.44; 2.6.1.45; 2.6.1.51]; *hprA*—glycerate reductase [EC 1.1.1.29]; *gck*—glycerate 2-kinase [EC 2.7.1.165]; *mdh*—malate dehydrogenase [EC 1.1.1.37]; *ppc*—phosphoenolpyruvate carboxylase [EC 4.1.1.31]; *mtkB*—malate-CoA ligase subunit alpha [EC 6.2.1.9]; *mtkA*—malate-CoA ligase subunit beta [EC 6.2.1.9]; *mcl*—malyl-CoA/(S)-citramalyl-CoA lyase [EC 4.1.3.24, 4.1.3.25]; *aceA*—isocitrate lyase [EC 4.1.3.1]; *aceB*—malate synthase G [EC 2.3.3.9]; *gph*—phosphoglycolate phosphatase [EC 3.1.3.18]; *glcD*—glycolate dehydrogenase FAD-linked subunit [EC 1.1.99.14]; *pgk*—phosphoglycerate kinase [EC 2.7.2.3]; *gapA*—NAD-dependent glyceraldehyde-3-phosphate dehydrogenase [EC 1.2.1.12]; *tpiA*—triosephosphate isomerase [EC 5.3.1.1]; *fbaA*—fructose-bisphosphate aldolase class II [EC 4.1.2.13]; *fbp*—fructose-1,6-bisphosphatase, type I [EC 3.1.3.11]; *tktA*—transketolase [EC 2.2.1.1]; *rpiA*—ribose-5-phosphate isomerase A [EC 5.3.1.6]; *rpe*—ribulose-phosphate 3-epimerase [EC 5.1.3.1]; *prk*—phosphoribulokinase [EC 2.7.1.19]; *rbcL*—ribulose bisphosphate carboxylase large chain [EC 4.1.1.39]; *rbcS*—ribulose bisphosphate carboxylase small chain [EC 4.1.1.39]. The complete annotation of enzyme genes involved in the pathways is provided in Table S1 (Supplementary Materials).

Genes of the Calvin–Benson–Bassham cycle were identified in the genomes of representatives of the genus *Sphaerotilus*. In all representatives in which the direct pathway of methanol oxidation via XoxF, the H_4_MPT pathway, and formate dehydrogenase was identified, all genes of the Calvin–Benson–Bassham cycle were found, including the key enzymes of the cycle, RuBisCO (*rbcLS*) and phosphoribulokinase (*prk*) (Figure 3).

In the genomes of representatives of the genus *Sphaerotilus*, genes encoding the key enzymes of the RuMP, 3-hexulose-6-phosphate synthase (*hxlA*) and 6-phospho-3-hexuloisomerase (*hxlB*), were not found, which catalyze the condensation of formaldehyde and ribulose 5-phosphate to hexulose 6-phosphate, followed by its conversion to fructose 6-phosphate (Figure 3).

Analysis of 20 genomes of the genus *Sphaerotilus* revealed the presence of several genes encoding enzymes of the serine cycle involved in the initial stage of C1 assimilation, such as serine–glyoxylate transaminase (*AGXT*), which participates in serine–glyoxylate, alanine–glyoxylate, and serine–pyruvate transamination reactions, and glycine hydroxymethyltransferase (*glyA*), which catalyzes the reversible conversion of serine and glycine with the participation of H_4_F. Additionally, the genomes were found to contain hydroxypyruvate reductase (*hprA*), which catalyzes the reduction of hydroxypyruvate to glycerate (Figure 3).

Representatives of the genus *Sphaerotilus* show a similar gene profile to the serine cycle, characterized by the absence of genes encoding malate-CoA ligase (*mtkBA*) and malyl-CoA lyase (*mcl*) (Figure 3). In the classical serine cycle, malate-CoA ligase (*mtkBA*) catalyzes the ATP-dependent activation of malate to form malyl-CoA, while malyl-CoA lyase (*mcl*) cleaves malyl-CoA to form glyoxylate and acetyl-CoA, thus allowing the regeneration of the C2 acceptor (glyoxylate) required for fixation of C1 compounds.

Thus, in terms of the functioning of the serine cycle, the identified set of enzymes allows the initial and intermediate stages of the pathway to proceed, leading to the formation of serine and subsequent metabolites, including phosphoenolpyruvate, pyruvate, and malate, which can be used in cellular biosynthetic processes. However, the absence of the *mtkBA* and *mcl* genes, which are responsible for malate conversion and the regeneration of glyoxylate required for subsequent condensation of methylene-H_4_F, suggests that the serine cycle cannot be completed in its classical form. It is likely that representatives of the genus *Sphaerotilus* lost the *mtkBA* and mcl genes during evolution, which led to the formation of an incomplete variant of the serine cycle.

Despite the incomplete serine cycle, regeneration of the C2 compounds is likely possible via the ethylmalonyl-CoA (EMC) pathway or the glyoxylate cycle [49]. Analysis of genes of the EMC pathway showed its absence in representatives of the genus *Sphaerotilus*. At the same time, genes encoding the key enzymes of the glyoxylate cycle (isocitrate lyase and malate synthase) are present in all analyzed genomes (Figure 3). However, its full functioning requires the activity of malyl-CoA lyase. Thus, the identified set of genes does not allow for a complete serine cycle in representatives of the genus *Sphaerotilus*.

However, genes encoding phosphoglycolate phosphatase (*gph*) and the FAD-dependent glycolate dehydrogenase subunit (*glcD*) were identified in the genomes of representatives of the genus *Sphaerotilus*. Their presence suggests a possible alternative pathway for the regeneration of glyoxylate from 2-phosphoglycolate, the product of the RuBisCO oxygenase reaction [50], which could support the operation of an incomplete serine cycle. Consequently, these findings suggest that representatives of the genus *Sphaerotilus* may retain the capacity for C1 assimilation through an incomplete serine cycle supported by alternative glyoxylate regeneration mechanisms.

### 2.2. Genomic Data Verification

To study methylotrophic growth in the genus *Sphaerotilus*, four species obtained in pure culture were used: *Sphaerotilus natans* DSM 6575^T^, *Sphaerotilus sulfidivorans* D-501^T^, *Sphaerotilus montanus* HS^T^, and *Sphaerotilus hippei* DSM 566^T^. In *S. sulfidivorans* D-501^T^, *S. montanus* HS^T^, and *S. hippei* DSM 566^T^, all genes of the Calvin–Benson–Bassham cycle were identified. Methanol dehydrogenase in *S. montanus* HS^T^ and *S. hippei* DSM 566^T^ is represented by two types: XoxF and Mdh2; genes of the H_4_MPT pathway of formaldehyde oxidation were also identified. In the genome of *S. sulfidivorans* D-501^T^, only the gene encoding the methanol dehydrogenase Mdh2 was identified, whereas formaldehyde oxidation is carried out via the H_4_F pathway.

#### 2.2.1. Increase in Protein Content in Sphaerotilus During Growth on Methanol

Analysis of the growth of the strains *S. sulfidivorans* D-501^T^, *S. montanus* HS^T^, and *S. hippei* DSM 566^T^ in the presence of methanol showed a stable increase in protein content during four passages. The average increase in protein content in these strains by the end of the exponential growth phase (72 h) was 16–20 mg protein L^−1^. The strain *S. natans* DSM 6575^T^ was used as a control, for which no growth on methanol was observed, even though the gene methanol dehydrogenase Mdh2 is present in its genome.

#### 2.2.2. Activity of Methanol Dehydrogenases and Formate Dehydrogenase

In the strain *S*. *sulfidivorans* D-501^T^, Mdh2-associated activity was detected only under methylotrophic conditions and was 0.034 ± 0.002 µmol min^−1^ mg^−1^ protein. In the strains *S. montanus* HS^T^ and *S. hippei* DSM 566^T^, methanol dehydrogenase activity associated with the presence of both XoxF and Mdh2 was observed only during methylotrophic growth and was 0.024 ± 0.002 and 0.020 ± 0.002 µmol min^−1^ mg^−1^ protein, respectively (Table 1). The activity of NAD-dependent formate dehydrogenase increased 3-4-fold in three strains under methylotrophic conditions compared to organoheterotrophic conditions and ranged from 0.021 to 0.030 ± 0.002 µmol min^−1^ mg^−1^ protein (Table 1).

#### 2.2.3. Activity of Carbon Fixation Enzymes, Phosphoribulokinase and Carbonic Anhydrase, During Methylotrophic Growth

Phosphoribulokinase activity was measured to demonstrate the functioning of the Calvin–Benson–Bassham cycle as the main pathway of CO_2_ fixation during methylotrophic growth. Carbonic anhydrase activity was measured to assess its role in maintaining a microenvironment with an increased CO_2_ concentration required for efficient RuBisCO carboxylation.

Phosphoribulokinase activity in the studied strains was observed only under methylotrophic conditions and ranged from 7.04 to 9.77 ± 0.02 µmol min^−1^ mg^−1^ protein. Carbonic anhydrase activity increased on average 4–5-fold under methylotrophic conditions compared to organoheterotrophic conditions and ranged from 12 to 14.5 Wilbur–Anderson units (Table 1) [51].

#### 2.2.4. Expression of *mdh2*, *xoxF*, *prk*, and *rbcL* Genes

To assess the activity of methanol-oxidizing pathways, the expression of the *mdh2* and *xoxF* genes was analyzed. In genomes in which these genes were not detected, transcriptional analysis was not carried out due to the absence of target sequences. The expression of the *rbcL* and *prk* genes was used as a marker of the functioning of the Calvin–Benson–Bassham cycle.

**Figure 4:**
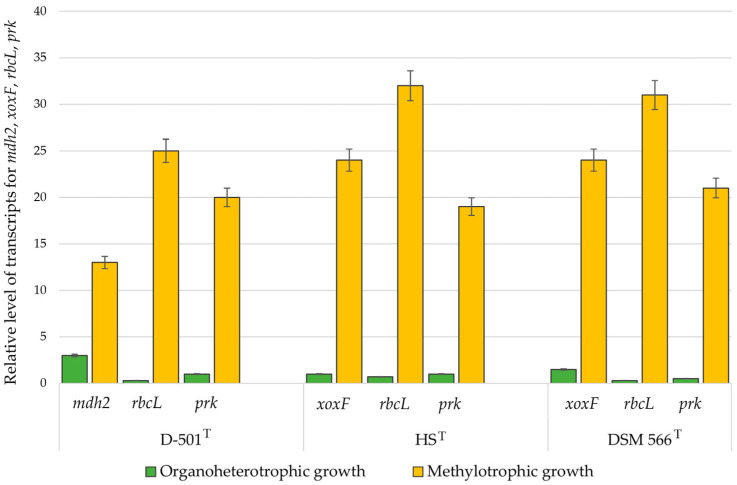
Expression of methylotrophic growth genes (*mdh2*, *xoxF*, *rbcL*, *prk*) in *S*. *sulfidivorans* D-501^T^, *S. montanus* HS^T^, and *S. hippei* DSM 566^T^.

The relative expression levels of the *mdh2*, *rbcL*, and *prk* genes in *S. sulfidivorans* D-501^T^ increased by 5-, 25-, and 83-fold, respectively, during growth on methanol compared to organoheterotrophic growth. For the strains *S. montanus* HS^T^ and *S. hippei* DSM 566^T^, the expression levels of the *xoxF*, *rbcL*, and *prk* genes increased on average by 15-, 29.9-, and 87.5-fold, respectively, compared to organoheterotrophic growth (Figure 4).

#### 2.2.5. Activity of Hydroxypyruvate Reductase, Isocitrate Lyase, and Glycolate Dehydrogenase

The activity of several enzymes of the serine cycle (hydroxypyruvate reductase) and the glyoxylate cycle (isocitrate lyase), and glycolate dehydrogenase, was analyzed to investigate pathways potentially contributing to C1 compound assimilation. Hydroxypyruvate reductase activity was detected only in *S. sulfidivorans* D-501^T^ and only under methylotrophic conditions, and was 0.04 ± 0.002 µmol min^−1^ mg^−1^ protein (Table 1). In *S. sulfidivorans* D-501^T^, isocitrate lyase activity under methylotrophic growth reached 0.24 ± 0.02 µmol min^−1^ mg^−1^ protein, which is 1.6-fold higher than under organoheterotrophic growth. In *S. montanus* HS^T^ and *S. hippei* DSM 566^T^, enzyme activity remained largely unchanged under different growth conditions and, under methylotrophic growth, was on average 12-fold lower than in *S. sulfidivorans* D-501^T^ (Table 1). Glycolate dehydrogenase activity was detected only under methylotrophic conditions in *S. sulfidivorans* D-501^T^ and was 0.24 ± 0.02 µmol min^−1^ mg^−1^ protein (Table 1).

## 3. Discussion

The carbon cycle is one of the major global biogeochemical topics of our time due to the intensification of the greenhouse effect. Methylotrophic microorganisms, along with methanotrophs, make a significant contribution to the biological transformation of carbon, participating in the maintenance of its balance in ecosystems.

Despite extensive data on methylotrophic microorganisms, the role of representatives of the genus *Sphaerotilus* in the transformation of C1 compounds has remained poorly studied to date.

Bioinformatic analysis of genomes of representatives of the revised genus *Sphaerotilus* revealed variability in methylotrophic strategies associated with differences in gene composition.

Two types of methanol dehydrogenases were identified in the genomes of the genus, XoxF and Mdh2. Notably, no complete MxaF methanol dehydrogenase systems were identified in any of the analyzed genomes. However, several genomes retained fragments of the *mxa*-cluster containing accessory and regulatory genes associated with MxaFI. Considering that some genomes retain individual components of the *mxa*-cluster, it is likely that in ancestral forms of representatives of the genus *Sphaerotilus*, the MxaFI system was functional, but during evolution, the genes encoding the catalytic subunits (*mxaF* and *mxaI*) were lost, while some accessory and regulatory genes were retained. Given that such cluster remnants were found in only a limited number of genomes (25%), reductive evolution of the MxaF system is more likely than its recent acquisition through horizontal gene transfer.

According to genome annotation data for representatives of the genus *Sphaerotilus*, the presence of genes of the tetrahydromethanopterin pathway is exclusively associated with the XoxF methanol dehydrogenase. In species whose genomes encode only the Mdh2 methanol dehydrogenase, formaldehyde oxidation proceeds via the tetrahydrofolate pathway. At the same time, the presence of the XoxF does not exclude the presence of both Mdh2 and the H_4_F pathway. This suggests the presence of alternative formaldehyde oxidation pathways in the cell, thus enabling optimization of metabolism when multiple systems are present concurrently.

Experimental data confirm the presence of methanol dehydrogenase activity in all studied strains during methylotrophic growth. Based on genome annotation data, the observed activity in *S*. *sulfidivorans* D-501^T^ is likely associated with Mdh2, whereas in *S. montanus* HS^T^ and *S*. *hippei* DSM 566^T^, it may reflect the presence of both XoxF and Mdh2. In addition, differences in the gene composition of formaldehyde oxidation pathways complement the observed enzymatic profile: the genome of *S. sulfidivorans* D-501^T^ lacks genes of the H_4_MPT pathway, indicating that formaldehyde oxidation proceeds exclusively via the H_4_F pathway, while in *S. montanus* HS^T^ and *S. hippei* DSM 566^T^, genes encoding both formaldehyde oxidation pathways are present.

Although methanol and formaldehyde oxidation demonstrate substantial variability among different species, the formate oxidation step remains conserved and is carried out by a single type of NAD-dependent formate dehydrogenase, the activity of which increases in the presence of methanol in all studied strains.

Assimilation of C1 compounds produced during direct methanol oxidation can occur via three metabolic cycles: the RuMP, the serine cycle, and the Calvin–Benson–Bassham cycle. In representatives of the genus *Sphaerotilus*, genes encoding the key enzymes of the RuMP cycle were not detected, while genes of the serine cycle (with the exception of the *mtkBA* and *mcl* genes) and a complete set of genes of the Calvin–Benson–Bassham cycle were identified.

Thus, representatives of the genus *Sphaerotilus* may use two strategies of carbon dioxide fixation. The first is based on the direct fixation of carbon dioxide via the complete Calvin–Benson–Bassham cycle, which enables CO_2_ assimilation and the synthesis of organic compounds independently of the serine cycle. The obtained data on enzyme activity and gene expression confirm the functioning of the Calvin–Benson–Bassham cycle during methylotrophic growth. Phosphoribulokinase activity was observed only in the presence of methanol, while carbonic anhydrase activity increased significantly, indicating the formation of conditions favorable for an efficient RuBisCO carboxylation reaction. In addition, the increased expression levels of the *rbcL* and *prk* genes during growth on methanol further support the functioning of the Calvin–Benson–Bassham cycle as one of the main pathways of CO_2_ fixation.

The second strategy is associated with the recovery of one of the functions of the serine cycle—the regeneration of the C2 compounds (glyoxylate) required for C1 assimilation. In this case, the incomplete serine cycle is maintained through the conversion of a product of the oxygenase activity of RuBisCO, 2-phosphoglycolate, into glyoxylate. This may be related to the fact that the set of genes and the combination of enzymatic pathways of direct methanol oxidation to carbon dioxide in these representatives are not optimal in terms of metabolic efficiency, and therefore the bacteria engage additional enzymatic capacities for more efficient C1 assimilation.

Biochemical and genomic data suggest that this strategy is primarily used by certain representatives of the genus *Sphaerotilus* lacking the XoxF and the H_4_MPT pathway. The methanol dehydrogenase Mdh2 and the H_4_F pathway of formaldehyde oxidation are characterized by lower activity and, consequently, reduced CO_2_ production during methylotrophic growth. This may reduce the local CO_2_ concentration required for the RuBisCO carboxylation reaction and shift the balance toward its oxygenase reaction, leading to the formation of 2-phosphoglycolate. Further conversion of 2-phosphoglycolate leads to the formation of glyoxylate, which enters reactions of the incomplete serine cycle and ensures the regeneration of C2 compounds.

Analysis of the genome of *S*. *sulfidivorans* D-501^T^ revealed only genes associated with the methanol dehydrogenase Mdh2 and the H_4_F pathway of formaldehyde oxidation. In addition, the increased activity of glycolate dehydrogenase may indicate enhanced glyoxylate formation, whereas the increased activity of hydroxypyruvate reductase is consistent with more active functioning of the incomplete serine cycle. Thus, in *S*. *sulfidivorans* D-501^T^, the incomplete serine cycle likely functions as an additional compensatory pathway of C1 assimilation in addition to the primary pathway via the Calvin–Benson–Bassham cycle.

In contrast, *S. montanus* HS^T^ and *S. hippei* DSM 566^T^ have a more efficient system of direct methanol oxidation to CO_2_, including XoxF and the H_4_MPT pathway. Enhanced CO_2_ accumulation promotes primarily the RuBisCO carboxylation reaction, thereby reducing the formation of 2-phosphoglycolate and, consequently, decreasing the involvement of the serine cycle in constructive metabolism. Under these conditions, carbon fixation occurs primarily via the Calvin–Benson–Bassham cycle.

**Figure 5:**
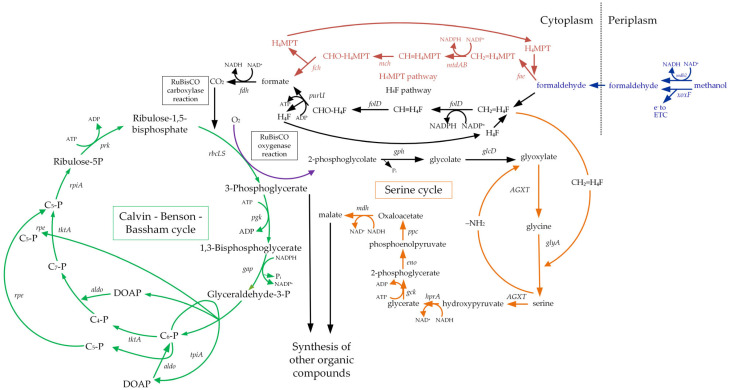
A hypothetical metabolic model of methylotrophic growth for the genus *Sphaerotilus*. Color code: green—Calvin–Benson–Bassham cycle; red—H_4_MPT pathway; black—H_4_F pathway, formate oxidation and 2-phosphoglycolate metabolism; purple—oxygenase reaction of RuBisCO; blue—oxidation of methanol to formaldehyde; orange—serine cycle. Detailed designations of genes are presented in Figure 1 and Figure 3.

Thus, in all studied strains, the Calvin–Benson–Bassham cycle functions as the main way of carbon fixation, while in some cases, mainly under local reductions in CO_2_ concentration and, consequently, limited RuBisCO carboxylation activity, the incomplete serine cycle may also be involved as a compensatory mechanism associated with the specific features of direct oxidation of methanol (Figure 5).

Metabolic flexibility in the way of CO_2_ fixation may contribute to bacterial survival under changing environmental conditions. It may be suggested that similar carbon assimilation strategies are also characteristic of other, as yet uncultivated, *Sphaerotilus* strains, consequently expanding current understanding of their ecological role.

## 4. Materials and Methods

### 4.1. Genome Annotation and Phylogenetic Analysis

Genome sequences of representatives of the genus *Sphaerotilus* were obtained from the NCBI GenBank database. Assembly statistics for the analyzed genomes are provided in Table S3 (Supplementary Materials). Identification and primary annotation of genes encoding proteins involved in direct methanol oxidation to CO_2_ and C1 fixation were carried out using the RAST server 2 [52]. The obtained annotations, including the identification of methanol dehydrogenase homologs, were refined by manual curation based on homology searches using BLASTP against the NCBI non-redundant (nr) protein database (http://blast.ncbi.nlm.nih.gov/Blast.cgi, accessed on 10 May 2025). Amino acid sequences were aligned using the ClustalW multiple sequence alignment algorithm implemented in MEGA 12 software (v.12.1.2). To determine the affiliation of the identified proteins and distinguish between MxaF- and XoxF-type methanol dehydrogenases, a phylogenetic analysis was performed using MxaF and XoxF proteins with experimentally confirmed functions. Phylogenetic tree was reconstructed using the minimum evolution method [53] based on p-distance and the maximum likelihood method based on the Jones–Taylor–Thornton substitution model [54] in MEGA 12 software (v.12.1.2) [55]. The reliability of the inferred topology was assessed by bootstrap analysis based on 1000 alternative trees.

### 4.2. Composition of Nutrient Media

Cultivation of the strains *Sphaerotilus natans* DSM 6575^T^, *Sphaerotilus sulfidivorans* D-501^T^, *Sphaerotilus montanus* HS^T^, and *Sphaerotilus hippei* DSM 566^T^ was carried out in a mineral medium of the following composition (g L^−1^): NH_4_Cl (0.30); K_2_HPO_4_ (0.021); KH_2_PO_4_ (0.0085); Na_2_HPO_4_ (0.034); MgSO_4_·7H_2_O (0.0225); CaCl_2_ (0.028) (https://www.dsmz.de/collection/catalogue/details/culture/DSM-22545, accessed on 21 September 2020, Medium 1664a). The pH of the medium was adjusted to 7.0 before inoculation using 1% NaOH and 10% HCl. Before cultivation, a vitamin solution containing thiamine (B1), riboflavin (B2), niacin (B3), pantothenic acid (B5), pyridoxine (B6), biotin (B7), folic acid (B9), and cobalamin (B12), as well as a trace element solution, was added to the medium [56]. The concentrations of the vitamin and trace element solutions were increased to 2 mL L^−1^ due to the increased demand of microorganisms for cofactors and their precursors during growth on methanol as a carbon source.

For methylotrophic growth, methanol was added to the medium at a concentration of 0.5 g L^−1^. Lanthanum chloride (LaCl_3_) (Himmedservice, Tver, Russia) was added to the cultivation medium at a concentration of 0.03 g L^−1^ to support the activity of the lanthanide-dependent methanol dehydrogenase XoxF. For cultivation in the presence of formate, sodium formate was added to the basic mineral medium at a concentration of 0.5 g L^−1^.

For organoheterotrophic growth, the cultivation medium was additionally supplemented with (g L^−1^): sodium lactate (0.30), glucose (0.30), and peptone (0.20).

The bacteria were incubated under aerobic conditions in flasks tightly sealed with rubber stoppers to limit gas exchange with the external environment.

### 4.3. Enzyme Activity Assays

The selected enzymes were used as markers of metabolic pathways involved in methylotrophic growth. Methanol dehydrogenase and formate dehydrogenase were assayed to verify methanol oxidation to CO_2_. Phosphoribulokinase and carbonic anhydrase were analyzed as indicators of Calvin–Benson–Bassham cycle activity, with carbonic anhydrase additionally evaluated for its role in maintaining a CO_2_-enriched microenvironment for efficient RuBisCO carboxylation. Hydroxypyruvate reductase served as a marker of the serine cycle, whereas isocitrate lyase and glycolate dehydrogenase were analyzed to assess the potential contribution of glyoxylate-generating pathways to C1 assimilation.

Formate dehydrogenase activity was determined spectrophotometrically by monitoring the increase in optical density at 340 nm resulting from NADH formation during formate oxidation. The reaction mixture contained 50 mM phosphate buffer (pH 7.0), 1.6 mM NAD^+^, and 0.3 M sodium formate. The reaction was initiated by the addition of cell-free supernatant (cytoplasmic fraction) [57].

Methanol dehydrogenase activity was determined spectrophotometrically by monitoring the decrease in optical density at 600 nm resulting from the reduction of 2,6-dichlorophenolindophenol (DCPIP). Measurements were performed using a modified method of Anthony and Zatman, 1964 [58]. The reaction mixture contained 300 mM Tris–HCl buffer (pH 9.0), 2.5 mM methanol, 3.3 mM phenazine methosulfate (PMS), 0.13 mM DCPIP, 45 mM NH_4_Cl, 10 mM CaCl_2_, and 50 mM potassium phosphate buffer (pH 7.5). The reaction was initiated by the addition of cell-free supernatant (cytoplasmic fraction). The modification of the original method included a reduced methanol concentration (2.5 mM instead of 20 mM), adjustment of the Tris–HCl buffer concentration, and the addition of CaCl_2_.

Phosphoribulokinase activity was determined by measuring phosphorus accumulation following alkaline hydrolysis of ribulose-1,5-bisphosphate. The reaction mixture contained 500 mM Tris–HCl buffer (pH 7.8), 50 mM MgCl_2_, 50 mM dithiothreitol, and 50 mM ribulose-5-phosphate (Merck KGaA, Darmstadt, Germany). The reaction was initiated by the addition of ATP and carried out at 30 °C [59].

Carbonic anhydrase activity was determined using the Wilbur–Anderson method by measuring the time required for the bromothymol blue indicator to change color from blue to yellow during accelerated CO_2_ hydration. The reaction mixture contained 25 mM Tris–HCl buffer (pH 8.2), 10 mg L^−1^ bromothymol blue, and cell-free supernatant (cytoplasmic fraction). The reaction was initiated by the addition of CO_2_-saturated cold water at 0 °C and carried out at 2 °C. Enzyme activity was expressed in Wilbur–Anderson units [51].

Isocitrate lyase activity was determined spectrophotometrically by measuring the formation of glyoxylate phenylhydrazone through monitoring the increase in optical density at 324 nm. The reaction mixture contained 70 mM Tris–HCl (pH 7.5), 1.9 M KCl, 5 mM MgCl_2_, 4 mM sodium isocitrate, and 4 mM phenylhydrazine hydrochloride. The reaction was initiated by the addition of cell-free supernatant (cytoplasmic fraction) [60].

Hydroxypyruvate reductase activity was determined spectrophotometrically by monitoring the decrease in optical density at 340 nm resulting from NADH oxidation during the reduction of hydroxypyruvate to glycerate. The reaction mixture contained 100 mM Tris–HCl buffer (pH 7.5), 0.67 mM hydroxypyruvate (Merck KGaA, Darmstadt, Germany), and 0.13 mM NADH. The reaction was initiated by the addition of cell-free supernatant (cytoplasmic fraction) [61].

Glycolate dehydrogenase activity was determined spectrophotometrically by monitoring the change in optical density at 324 nm. The reaction mixture consisted of 100 µmol Tris–HCl buffer (pH 8.0), 0.2 µmol DCPIP, 0.1 mL of 1% (*w*/*v*) PMS, 10 µmol potassium glycolate, and 0.1 M phenylhydrazine hydrochloride was added. The reaction was initiated by the addition of the cell-free supernatant (cytoplasmic fraction) [62].

### 4.4. Gene Expression Analysis

Gene expression was analyzed during methylotrophic growth of *S*. *sulfidivorans* D-501^T^, *S*. *montanus* HS^T^, and *S*. *hippei* DSM 566^T^, while organoheterotrophic growth was used as a control. The 16S rRNA gene (*rrs*) was used as the reference gene for analysis.

RNA was isolated using the ExtractRNA reagent (Evrogen, Moscow, Russia) in accordance with the manufacturer’s protocol. RNA quality was evaluated by electrophoresis on a 1.1% agarose gel with 2.2 M formaldehyde added. RNA concentration was measured using an HS Equalbit RNA assay kit (Vazyme, Nanjing, China) on a Fluo-200 fluorometer (Allsheng, Hangzhou, China). Then, 2000 ng of RNA was reverse transcribed using M-MulV (SybEnzyme, Moscow, Russia) according to the manufacturer’s protocol. RT-qPCR was performed using SYBR Green I on a CFX96 Touch Real-Time PCR Detection System (Bio-Rad, Hercules, CA, USA).

A temperature gradient was used to determine the optimal amplification conditions. The amplification program for the *rrs*, *xoxF*, *mdh2*, *prk*, and *rbcL* genes included 95 °C for 5 min followed by 30 cycles of 95 °C for 10 s, 60 °C for 20 s, and 72 °C for 15 s. The primers used in this study are listed in Table S4 (Supplementary Materials). All primers were designed using PrimerBLAST (http://www.ncbi.nlm.nih.gov/tools/primer-blast, accessed on 8 November 2025).

## 5. Conclusions

Representatives of the genus *Sphaerotilus* are widely distributed in aquatic ecosystems and are characterized by flexible metabolism and the ability to occupy diverse ecological niches. Recent genomic data have significantly expanded the current understanding of their physiological potential, indicating the diversity of the metabolic strategies they employ. However, before this study, information on the autotrophic and methylotrophic metabolism of representatives of the genus remained extremely limited.

The results of genome annotation obtained in this study and their experimental verification substantially expand the current understanding of carbon transformation pathways in *Sphaerotilus*, despite the still limited number of available pure cultures, while their cosmopolitan status highlights the ecological relevance of these findings. In this context, the analysis of C1 transformation pathways makes it possible to better define the role of representatives of the genus in the biogeochemical carbon cycle.

Representatives of the genus *Sphaerotilus* demonstrate variability in methylotrophic growth pathways depending on gene composition and environmental conditions. At the same time, carbon fixation in all studied strains occurs primarily via the Calvin–Benson–Bassham cycle as the main way of CO_2_ assimilation, whereas the incomplete serine cycle may be involved as a compensatory mechanism under reduced local CO_2_ availability and limited RuBisCO carboxylation activity.

Studies of methylotrophy in the genus *Sphaerotilus* and further understanding of C1 compound metabolism will help answer a number of questions related to the ecology and geographic distribution of these bacteria, as well as their role in the detoxification of toxic compounds, and may contribute to the development of approaches for controlling their active growth in industrial systems or, conversely, increasing the yield of feed protein or target products in biotechnology.

## Figures and Tables

**Table 1:** Enzyme activities associated with methylotrophic growth in the genus *Sphaerotilus*. Activity values are presented as mean ± SD from three biological replicates, each measured in triplicate. n/o—no activity detected.

Metabolic Process	Pathway	Enzyme	Growth Conditions	Enzyme Activity, µmol min^−1^ mg^−1^ Protein			
				***S. sulfidivorans*** D-501^T^	***S. montanus*** HS^T^	***S. hippei*** DSM 566^T^	***S. natans*** DSM 6575^T^
Methanol oxidation	Methanol → Formaldehyde	NAD-dependent methanol dehydrogenase Mdh2	Organoheterotrophic growth	n/o	n/o	n/o	n/o
			Methylotrophic growth	0.034 ± 0.002	0.024 ± 0.002	0.020 ± 0.002	n/o
		PQQ-dependent methanol dehydrogenase XoxF	Organoheterotrophic growth	n/o	n/o	n/o	n/o
			Methylotrophic growth	n/o	0.024 ± 0.002	0.020 ± 0.002	n/o
	Formate → CO_2_	Formate dehydrogenase	Organoheterotrophic growth	0.006 ± 0.001	0.008 ± 0.001	0.009 ± 0.001	0.011 ± 0.001
			Methylotrophic growth	0.021 ± 0.002	0.030 ± 0.002	0.029 ± 0.002	n/o
C1 compound assimilation	Calvin–Benson–Bassham cycle	Phosphoribulokinase	Organoheterotrophic growth	n/o	n/o	n/o	n/o
			Methylotrophic growth	8.36 ± 0.02	9.77 ± 0.02	7.04 ± 0.02	n/o
		Carbonic anhydrase *	Organoheterotrophic growth	2.0 ± 0.05	2.3 ± 0.05	3.0 ± 0.05	n/o
			Methylotrophic growth	12.0 ± 0.05	14.0 ± 0.05	14.5 ± 0.05	n/o
	Serine cycle	Hydroxypyruvate reductase	Organoheterotrophic growth	n/o	n/o	n/o	n/o
			Methylotrophic growth	0.04 ± 0.002	n/o	n/o	n/o
	Glyoxylate cycle	Isocitrate lyase	Organoheterotrophic growth	0.14 ± 0.02	0.15 ± 0.02	0.13 ± 0.02	0.134 ± 0.02
			Methylotrophic growth	0.24 ± 0.02	0.02 ± 0.002	0.022 ± 0.002	n/o
	Glyoxylate biosynthesis	Glycolate dehydrogenase	Organoheterotrophic growth	n/o	n/o	n/o	n/o
			Methylotrophic growth	0.24 ± 0.02	n/o	n/o	n/o

* Carbonic anhydrase activity was measured in Wilbur–Anderson units.

## Data Availability

The original contributions presented in this study are included in the article/Supplementary Material. Further inquiries can be directed to the corresponding author.

## Supplementary Materials

The following supporting information can be downloaded at: https://www.mdpi.com/article/10.3390/ijms27125498/s1.
