# Supplementary material for: Comparative Genomic Analysis and Data About the Metabolism of the Genus Sphaerotilus Provide the First Evidence of Methylotrophic Growth and Reveal Two Strategies of Methanol Oxidation and C1 Compound Assimilation
Source: Int J Mol Sci. 2026 Jun 18;27(12):5498. doi: 10.3390/ijms27125498 (PMC13300288; doi:10.3390/ijms27125498)
Supplement: Supplementary file 1 [file ijms-27-05498-s001.zip › Supplementary Materials.pdf]

Table S2. Protein accession numbers of reference methanol dehydrogenases used for the phylogenetic analysis (Wu et al., 2015; Daszczyńska et al., 2022).

Names of methanol dehydrogenase protein types	Protein sequence accession numbers
PQQ-dependent methanol dehydrogenase XoxF1	WP_023786075.1, WP_020176222.1, WP_026867884.1, WP_019899159.1, WP_020494391.1, ANB17083.1, WP_006451751.1, WP_010341918.1
PQQ-dependent methanol dehydrogenase XoxF2	CBE67248.1, AAY96669.1, ABX56636.1, ACM16493.1
PQQ-dependent methanol dehydrogenase XoxF3	ABE49719.1, WP_011688545.1, ABR64943.1, WP_012745854.1, WP_014239620.1
PQQ-dependent methanol dehydrogenase XoxF4	WP_019897221.1, WP_020182652.1, WP_055825434.1, ABE49985.1, WP_015830880.1, WP_013149133.1, EAV46784.1, WP_013147907.1
PQQ-dependent methanol dehydrogenase XoxF5	WP_011490928.1, WP_045780266.1, WP_014149914.1, WP_002682507.1, ABS67026.1, WP_012045858.1, WP_011746370.1, WP_011567180.1
PQQ-dependent methanol dehydrogenase MxaF	AAG49450.1, P16027, P15279.1, BAE16958.1, CBE67228.1, AAA83765.1, AAD56237.2

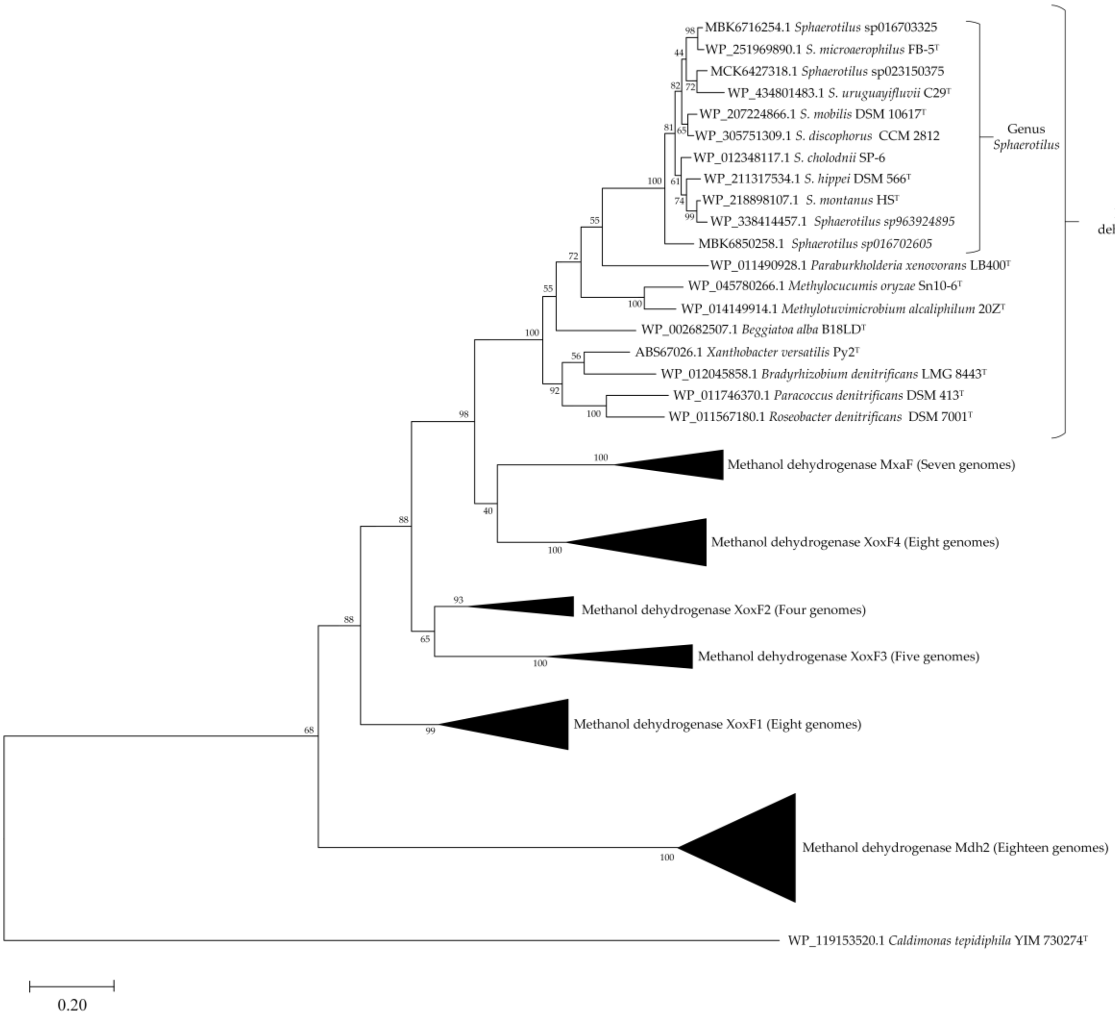

Figure S1. Phylogenetic tree of *Sphaerotilus* methanol dehydrogenases based on MxaF, XoxF1–5, and Mdh2 protein sequences constructed using the maximum-likelihood method based on the Jones–Taylor–Thornton substitution model (Jones et al., 1992). Mdh2 proteins detected in *Sphaerotilus* were included for completeness and formed a distinct clade separate from XoxF proteins. Numbers at the branch nodes indicate bootstrap values expressed as percentages of 1000 replicates. The phylogenetic analysis included reference sequences with previously established phylogenetic positions and functional characteristics (Wu et al., 2015; Daszczyńska et al., 2022). The tree was rooted using the alcohol dehydrogenase of *Calidimonas tepidiphila* YIM 73027^{T} (WP_119153520.1) as an outgroup. The scale bar represents 0.2 substitutions per site. A complete list of reference proteins from other bacterial species used for tree construction is provided in Table S2.

Table S3. General characteristics of genomes taken from the NCBI GenBank databases (Smolyakov et al., 2025).

Genome No	GTDB species name	Genome Assembly	Isolate or MAG	Size (MB)	Contigs	Completeness (%)	Contamination (%)
1	*Sphaerotilus* sp016703325	GCA_016703325.1	MAG	5,6	76	93.32	2.26
2	*Sphaerotilus* sp023150375	GCA_023150375.1	MAG	4,6	43	100	0.08
3	*Sphaerotilus microaerophilus*	GCA_023734135.1	MAG	6,05	2	99.99	0.22
4	*Sphaerotilus cholodnii*	GCA_000019785.1	Isolate	4,9	1	99.99	0.14
5	*Sphaerotilus mobilis*	GCA_004216565.1	Isolate	4,6	15	99.99	0.15
6	*Sphaerotilus discophorus*	GCF_030705305.1	Isolate	4,6	33	100	0.08
7	*Sphaerotilus hippei*	GCA_003201595.1	Isolate	4,4	46	100	0.04
8	*Sphaerotilus* sp034519685	GCF_034519685.1	MAG	5,02	7	99.99	0.98
9	*Sphaerotilus montanus*	GCA_013426955.1	Isolate	4,9	203	100	0.05
10	*Sphaerotilus* sp963924895	GCF_963924895.1	MAG	4,4	71	97.61	1.47
11	*Sphaerotilus* sp041083715	GCF_041083715.1	MAG	3,9	420	91.7	2.34
12	*Sphaerotilus* sp031429695	GCF_031429695.1	MAG	4,5	112	100	0.89
13	*Sphaerotilus* sp013299175	GCF_013299175.1	MAG	3,9	201	94.3	1.89
14	*Sphaerotilus uruguayifluvii*	GCA_013294065.1	Isolate	4,8	48	99.99	0.05
15	*Sphaerotilus sulfidivorans*	GCA_013426975.1	Isolate	4,3	126	99.96	0.1
16	*Sphaerotilus natans*	GCA_000689195.1	Isolate	4,5	188	99.93	0.23
17	*Sphaerotilus* sp041083665	GCA_041083665.1	MAG	4,6	66	99.99	1.91
18	*Sphaerotilus ochracea*	GCF_041083635.1	MAG	2,9	54	100	0
19	*Sphaerotilus* sp016702605	GCA_016702605.1	MAG	5,9	24	97.96	1.05
20	*Sphaerotilus* sp041169165	GCA_041169165.1	MAG	5,3	102	99.98	1.54

Table S4. Primers used to study methylotrophic growth in representatives of the genus *Sphaerotilus*.

Target gene	Primer	Primer sequence, 5′–3′	Tm, °C
		S. sulfidivorans D-501ᵀ	
rrs	Forward	ACTTCACCCCAGTCACGAAC	59.89
	Reverse	AATACGTAGGGTGCAAGCGT	59.75
mdh2	Forward	CGAACATCTTGCCGTTGTGG	60.11
	Reverse	CGCTATTCCCCGCTGAAGAA	60.18
rbcL	Forward	CCATCTGGCGGTACTTGAGG	60.18
	Reverse	ATGAACCAGCCCGTCGAAC	60.38
prk	Forward	GGAAAGTCGATGCCCTTCGG	61.09
	Reverse	CGCATGTCAACTTCCAGCG	59.87
		S. montanus HSᵀ	
rrs	Forward	TACAGATCGCAGGCTTGGTG	60.11
	Reverse	AATACCGCATACGACCCGAG	59.69
xoxF	Forward	GAACTTCTCGGCCTTGCTCT	60.04
	Reverse	TTCAAGGTCGAGTACACCGC	60.04
rbcL	Forward	AGGAGTGGTACGGCATGAAG	59.46
	Reverse	CAGCGGTGTTGATGACGTTG	60.11
prk	Forward	ACGTTCTTGTCGCGGTAGAG	60.11
	Reverse	CACACCGACCTGCTGTTCTA	59.68
		S. hippei DSM 566ᵀ	
rrs	Forward	GCAGCAGTGGGGAATTTTGG	60.04
	Reverse	GCAGTTTACAACCCGAAGGC	59.76
xoxF	Forward	TTCAAGGTCGAGTACACCGC	60.04
	Reverse	GAACTTCTCGGCCTTGCTCT	60.04
rbcL	Forward	AGGCGTAGTTGAAGGACACG	60.04
	Reverse	CGCTGGAATGCATGGTCAAG	59.90
prk	Forward	TCATGCTCAGCAGGTAGGGA	60.33
	Reverse	ACACCAGCAACCCCTTCATC	60.25
